# Transcutaneous electrical nerve stimulation for the management of tennis elbow: a pragmatic randomized controlled trial: the TATE trial (ISRCTN 87141084)

**DOI:** 10.1186/1471-2474-10-156

**Published:** 2009-12-11

**Authors:** Linda S Chesterton, Daniëlle A van der Windt, Julius Sim, Martyn Lewis, Christian D Mallen, Elizabeth E Mason, Catherine Warlow, Kanchan Vohora, Elaine M Hay

**Affiliations:** 1 Arthritis Research Campaign National Primary Care Centre, Keele University, Staffordshire, ST5 5BG, UK

## Abstract

**Background:**

Tennis elbow is a common and often extremely painful musculoskeletal condition, which has considerable impact on individuals as well as economic implications for healthcare utilization and absence from work. Many management strategies have been studied in clinical trials. Whilst corticosteroid injections offer short term pain relief, this treatment is unpleasant and is used with caution due to an associated high risk of pain recurrence in the long term. Systematic reviews conclude that there is no clear and effective treatment for symptoms of pain in the first 6 weeks of the condition. There is a clear need for an intervention that is acceptable to patients and provides them with effective short-term pain relief without increasing the risk of recurrence. Transcutaneous electrical nerve stimulation (TENS) is an inexpensive, non-invasive, non-pharmacological form of analgesia that is commonly used in the treatment of pain. TENS has very few contraindications and is simple to apply. It also benefits from being patient controlled, thereby promoting self-management. This study aims to assess the effectiveness, in terms of pain relief, and cost-effectiveness of a self-management package of treatment that includes TENS.

**Methods/Design:**

The design of the study will be a two-group pragmatic randomized clinical trial. 240 participants aged 18 years and over with tennis elbow will be recruited from 20-30 GP practices in Staffordshire, UK. Participants are to be randomized on a 1:1 basis to receive either primary care management (standard GP consultation, medication, advice and education) or primary care management with the addition of TENS, over 6 weeks. Our primary outcome measure is average intensity of elbow pain in the past 24 hours (0-10 point numerical rating scale) at 6 weeks. Secondary outcomes include pain and limitation of function, global assessment of change, days of sick leave, illness perceptions, and overall health status. A cost-effectiveness analysis will also be performed. Patient adherence and satisfaction data will be collected at 6 weeks, 6 months and 12 months by postal questionnaire. A diary will also be completed for the first 2 weeks of treatment. Clinical effectiveness and cost-effectiveness analyses will be carried out using an intention-to-treat approach as the primary analysis.

**Discussion:**

This paper presents detail on the rationale, design, methods and operational aspects of the trial.

**Trial registration:**

Current Controlled Trials. ISRCTN87141084

## Background

Tennis elbow is a painful, disabling musculoskeletal condition predominant in the 35-50 age group, and often causes considerable pain in normal daily activities such as gripping, carrying and lifting. Tennis elbow is traditionally considered to be self-limiting, but may last for 6-18 months [1]. Its estimated prevalence in the general population is 3-7% [2]. However, workers undertaking repetitive tasks are at greater risk, representing between 35-64% of all cases [3].

More than 40 treatments have been proposed for tennis elbow, some of which have been investigated in clinical trials and systematic reviews [4-6]. The overall conclusion of these studies is that there is insufficient evidence that any of these treatments are effective, either in the long or short term. A recent study by Bissett et al [7] investigated the effects of physiotherapy, corticosteroid injection, or a 'wait-and-see' approach. This investigation was similar to two previous trials [8,9]. The results from these trials showed dramatic short-term pain relief from corticosteroid injection, but poor long-term results, with higher recurrence rate compared with the non-injection groups. In the trial by Bisset et al [7], although physiotherapy produced slightly better results than 'wait-and-see' at six weeks, it was not as effective for pain relief as injections. Furthermore, from the patients' perspective, corticosteroid injections are seen as unpleasant and are associated with initial worsening of pain [10]. Therefore, in the first 6-12 weeks, there is still no clear treatment for patients who experience considerable pain and reduced function. There is a need for an alternative intervention that provides acceptable, effective short-term pain relief without increasing the risk of long-term recurrence.

Transcutaneous electrical nerve stimulation (TENS) is an inexpensive, safe, non-pharmacological form of analgesia. TENS units can be used in various clinical settings, are economically priced, and can be readily purchased by patients. TENS is simple to apply and is patient controlled, promoting self-management. The neurophysiological basis for pain relief from electrical stimulation such as TENS derives directly from the 'gate control' theory of pain [11]. TENS has shown positive analgesic effects in seven systematic reviews across a range of clinical conditions, e.g. chronic musculoskeletal pain [12], knee osteoarthritis [13] and rheumatoid arthritis [14]. Furthermore, a recent meta-analysis on postoperative use of TENS [15] showed that a dose-dependent effect is clearly observable, and when TENS is applied with an evidence-based dose, analgesic consumption could be reduced by up to 35%. Long-term follow up of patients using TENS has shown a 40-58% reduction in pain [16], coupled with a reduction in interference with work, domestic, and social activities, increased activity levels, and decreased use of other therapies/medication [17].

It has been argued that non-specific (placebo-related) factors of TENS comprise much of the total response. However experimental laboratory studies have clearly demonstrated the efficacy of TENS [18,19], and clinical trials have also shown beneficial effects compared with sham TENS interventions [15]. Those systematic reviews of TENS producing negative (*n *= 3) or inconclusive results (*n *= 4) [20] included primary studies that used inadequate TENS dose and were underpowered [12].

Only two previous studies have investigated TENS in tennis elbow. Halle et al [21] showed a decrease in mean pain intensity after 5 days, but the brief treatment period and small sample (*n *= 12 per group) restrict robust inferences. Weng et al [22] showed a positive outcome for TENS and a reduction in pain, but utilized non-industry standard TENS and the sample size (*n *= 20 per group) was again small.

### Aims and purpose of the proposed research

The overall aim of this trial is to investigate if TENS, as a patient-controlled adjunct to routine primary care for tennis elbow, can provide superior short term pain relief and functional improvement compared with routine primary care alone, without increasing the risk of long-term recurrence.

The primary objective of the trial is to investigate, at six weeks, pain relief from a self-management package of treatment that includes TENS in addition to primary care management (analgesia with advice and information regarding tennis elbow self management), compared with primary care management alone, in patients presenting to their general practitioner (GP) with a first or new clinical diagnosis of tennis elbow.

The secondary objectives of the trial are to investigate:

• pain relief from TENS self-management at 6 and 12 months compared with primary care management alone.

• differences in secondary outcomes (participation in work and other usual activities, use of analgesics, limitation in function, and illness perceptions) between TENS self-management and primary care management alone, at each time point.

• differences in symptom recurrence between TENS self-management and primary care management alone, at 12 months.

• the cost-effectiveness of TENS self-management as an addition to primary care management.

• the influence of process measures, in particular patient adherence, expectations, and satisfaction with treatment.

## Methods/Design

**Figure 1:**
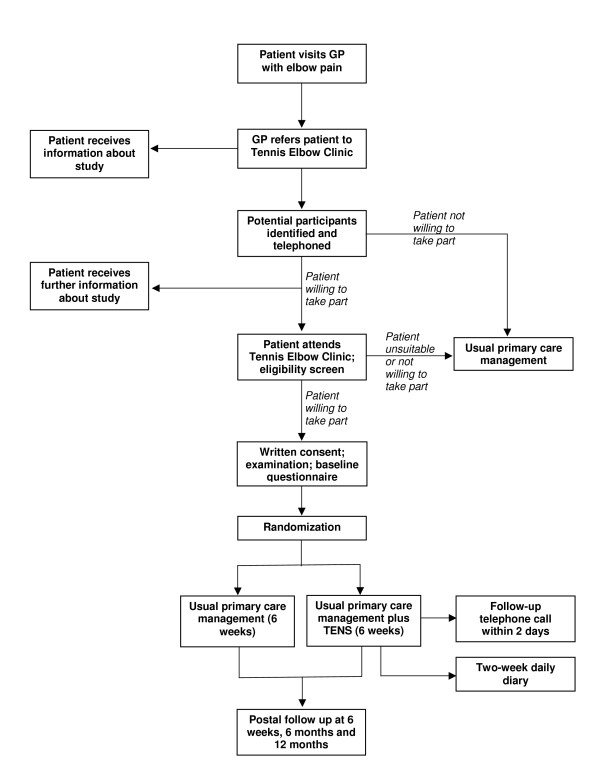
**Trial flow chart**.

The design of the study will be a two-group pragmatic randomized clinical trial; this is illustrated in Figure 1.

### Setting and recruitment of participants

Participants will be recruited from 20-30 GP practices in Staffordshire, England. The area covered by these practices is primarily urban with some rural and inner city areas, and is therefore broadly representative of the population in the UK.

#### Eligibility criteria

Male and female subjects aged 18 years and above who consult their GP with a first or new clinical diagnosis of tennis elbow (i.e. adults with pain and tenderness in the lateral region of the elbow, increasing on pressure on the lateral epicondyle and on resisted dorsiflexion of the wrist) are eligible to take part.

Participants must speak and understand English and be willing and able to give informed written consent. Exclusion criteria for the trial are: history of inflammatory arthritis or gross structural abnormality of the elbow; contraindications to TENS (pacemakers, epilepsy, dermatological conditions, abnormal sensation in the affected arm, indwelling electrical pumps/pacemakers, and pregnancy); neuropathic pain; inability to apply TENS independently, complete written questionnaires, or read instruction leaflets written in English.

#### Recruitment

GPs who are networked on the EMIS data system (a computerized system that maintains electronic patient records) will enter an appropriate diagnostic code (Read Code) for tennis elbow, and a 'pop-up' computer prompt will remind the GP that the patient is eligible for inclusion in the trial. At this point the GP will have the facility to enter a 'flag' onto the computer system if patients are unsuitable for the trial. A similar system to recruit patients to previous musculoskeletal trials in primary care has been used in this Centre [23]. GPs who are not networked on the EMIS system will be provided with a manual prompt card regarding eligibility for the trial and exclusion criteria. Suitable patients will be told about the trial and the Tennis Elbow Clinic. Interested patients will be given an information leaflet about the trial and the GP will then obtain written consent to contact by a study nurse, which will be faxed to the research centre along with the patient's details.

All patients who consent to further contact by a study nurse will then receive treatment by the GP, including analgesic prescription (where appropriate) and/or advice (e.g. paracetamol or co-codamol up to 8 tablets per day). Patients receiving prescribed anti-inflammatory or pain-relieving medication will be permitted to continue a stable dose. GPs will be asked to avoid corticosteroid injections or referral to physiotherapy for the first 6 weeks of the patient's participation in the trial.

The study nurse will telephone patients within two working days to provide further information about the trial, establish that the patient has not received steroid injection or referral to physiotherapy from the GP and is eligible for the trial, and make an appointment for the Tennis Elbow Clinic. Patients will be notified that the consultation may take up to an hour. They will then be sent a letter to confirm their appointment, information about the clinic, and a duplicate trial information leaflet. If the appointment is within 24 hours, oral instructions about the clinic will be given by the study nurse.

At the Tennis Elbow Clinic the study nurse will welcome patients and check their details. The study nurse will then explain the purpose of the clinic and the research being hosted within the clinic, check eligibility, and ask patients whether they wish to take part in the trial. Patients who are not eligible or do not wish to take part in the trial will be seen by a second treatment clinician (physiotherapist or nurse) and given information and advice on managing their tennis elbow according to usual practice. For patients giving preliminary consent, the study nurse will explain the trial in detail, gain written informed consent including permission for medical record review. The patient will then be provided with a baseline questionnaire to complete. The study nurse will check the baseline questionnaire for completeness, prior to randomization. The study nurse will remain blinded to treatment allocation throughout the trial. A post-study audit of the success of blinding procedures will be completed and a procedure for reporting incidents where blinding has been compromised will be in place.

#### Baseline questionnaire

In addition to baseline outcome measurements, as appropriate, the baseline questionnaire will include:

• Sociodemographic variables

• Work-related variables: hours of paid work, job type, physical work load (items from questionnaire developed by Bot et al [24]), psychosocial work environment (items from Job Content Questionnaire [25]).

• Clinical characteristics: history of elbow pain, previous treatments, intensity and duration of symptoms, other co-existing pain problems.

• Beliefs and expectations regarding treatment: items of the revised Illness Perceptions Questionnaire (IPQ-R) [26] supplemented with specific questions on treatment expectations

• Illness perceptions (including personal control and expectations regarding duration, items from IPQ-R)[26].

### Randomization and Treatment Allocation

After completing baseline assessment consenting patients will be randomly allocated in a 1:1 ratio to six weeks of either primary care management alone or primary care management with TENS. Prior to the start of the trial, the trial coordinator, who is not involved in the selection and inclusion of patients, will supervise preparation of numbered, opaque and sealed tamper-proof envelopes containing the treatment allocation. The random sequence (based on simple randomization) will be generated using a computerized random-number generator. At the research clinic, after final enrolment and baseline assessment, the participant is allocated an individual study number, and receives the appropriate envelope from the research nurse. The participant then meets with the treating clinician, and the envelope is opened. The use of separate study and treating clinicians and sealed envelope randomization will ensure independent selection of patients and concealed allocation of treatment.

Following randomization, the treating clinician will deliver whichever intervention the patient has been randomized to receive. The clinician will also give each patient a two-week daily diary to measure pain intensity and record usage of TENS and analgesics. Patients from both treatment arms will be advised that they can access their GP for ongoing care in the usual way if their elbow pain becomes worse.

Patients will be sent postal questionnaires at 6 weeks, 6 months and 12 months, with reminder letters 2 and 4 weeks following first mail-out for those who do not respond. The study nurse will telephone patients who do not respond to the first reminder letter at six week follow up or the second reminder letter at six- or twelve-month follow up, to collect minimum data on the primary outcome.

### Interventions

#### Primary care management alone (control intervention)

At the research clinic the treatment clinician will provide patients with the tennis elbow information leaflet and will reinforce the messages on advice and education contained within it. Patients will be informed of the usually self-limiting nature of tennis elbow and advised that while the elbow pain persists, they should avoid, whenever possible, repetitive elbow extension, forceful elbow activities, or activities that provoke pain. Potential ergonomic impact factors derived from sporting or working activities will be discussed and self-management in the form of rest/avoidance suggested, although absolute rest of the arm will not be advocated. In addition, the patient will be advised to gradually increase activity once acute pain has settled down and some basic progressive exercises will be explained.

#### Primary care management plus patient-controlled TENS

In addition to the control intervention described above, patients randomized to this group will be given a TENS machine and instructed on how to use it. They will be shown how to apply TENS locally, to the lateral aspect of the elbow and forearm, and will be encouraged to use the TENS machine at least once per day for one 45-minute treatment session, for each day that symptoms persist. Patients may use the TENS machine more often if they wish. The TENS parameter settings will be high frequency (110 Hz) at a pulse duration of 200 μs (frequency and pulse duration will be pre- programmed), with a self-selected intensity described as of 'strong but tolerable sensation' (measured as amplitude mA). Patients will be informed that they should feel an uncomfortable (but not painful) 'tingling' sensation and that they may experience muscle contractions and a local cooling of the area. Patients will be encouraged to use the TENS machine for a minimum of six weeks unless their symptoms have fully resolved before then.

The selected TENS settings are those that, on the basis of the literature and our pilot laboratory work, are most likely to achieve analgesia. Patients will be required to rest during, and for approximately one hour after, the stimulation period, which will last 45 minutes. Application just before usual bedtime will be recommended so that the rest period can be incorporated into normal rest/sleep periods. An exact timing of stimulation is not essential provided that participants adhere to these conditions. The application of TENS will be the responsibility of the patient but the treating clinician will make an appointment for a telephone consultation approximately two days after the clinic to review the technical application of TENS and will be available via telephone (during the six-week treatment period) to assist patients with technical queries.

### Training of the treatment clinicians

Prior to recruitment of participants into the trial, the study treating clinicians will be trained to deliver the interventions in a standardized way. Training will include information regarding the clinical syndrome of tennis elbow, presenting symptoms, and treatment options. Guidance in the delivery of specific advice, in respect of work and ergonomic factors and exercises outlined in the leaflet, will also be given. Specific training in TENS will include: how it works, how it should be applied, how to check for contraindications and precautions, and how to train patients in its use. Technical difficulties with the TENS units are unlikely to occur but training will also be provided in fault finding in order to support patients in their use of TENS at their follow-up phone call. The core training will be covered in one day with a further day's training two weeks later, just prior to the start of the clinics, to practise delivering the interventions. A written manual of instructions on how to deliver the interventions will be given to each clinician.

### Audit of interventions

At the clinic, the study clinician will complete a case report form for each patient participating in the trial; this will record assessment findings relating to elbow pain and document advice and treatment given in clinic. Ten percent of these forms will be audited, and members of the study team will also visit the clinic periodically to ensure the appropriate delivery of advice and treatment.

### Equipment

The TENS units to be used (TensCare itouch Easy™) will be pre-set with the stimulation parameters. Patients will be given basic coaching in use of the equipment - e.g. switching on and off and changing the battery. However, patients will not be instructed on how to change programmes within the machine and will be asked to ensure they do not attempt to do this.

### Outcome Measures

Clinical outcomes will be measured at baseline in clinic and by postal questionnaire at six weeks, six months and 12 months. There will also be a daily diary issued at clinic to be completed by participants over the first two weeks of treatment and returned by post on completion. The diary for the participants receiving TENS will have additional questions to record daily use of the TENS machine. Specific content is as follows:

• Intensity of elbow pain in the past 24 hours (0-10 point Numerical Rating Scale, NRS);

• Sick leave or inability to carry out usual activities due to elbow pain;

• Number and type of analgesics taken per day;

• The use of TENS in minutes per day (for the TENS group).

#### Questionnaires at 6 weeks, 6 months and 12 months after randomization

All participants will receive a postal questionnaire containing primary and secondary outcome measures. As the main focus of the trial is reduction of pain in patients with tennis elbow the primary outcome will be the average intensity of elbow pain over the past 24 hours scored on a 0-10 NRS scale. Secondary outcomes will be:

• Days sick leave and ability to carry out usual activities

• Self reported global change in elbow pain (5-point adjectival scale: much better - much worse [9])

• Pain and limitation in function (Patient-rated Tennis Elbow Evaluation (PTEE) [27])

• Illness perception (items in the IPQ-R [26])

• General Health: the EuroQoL EQ-5D [28] and SF-12 SF-6D [29].

• Health care resource use (including visits to health care professionals, and use of co-interventions and analgesics)

• Process measures:

◦ Changes in beliefs and expectations of treatment (IPQ-R and specific items)

◦ Satisfaction with treatment, feasibility, and applicability of TENS (0-10 NRS)

◦ Compliance with treatment (adherence to advice/exercise, and use of TENS in minutes per day in the TENS group).

### Sample size

In two previous trials of tennis elbow in primary care in the UK and the Netherlands, there was a 25% reduction in pain (1.5-point mean change (standard deviation 2.6) on a 0-10 NRS) in the usual care group at 4-6 weeks follow-up [8,9]. The overall mean baseline pain score was 6-points in both trials (after standardizing to a 0-10 point scale). According to Farrar et al [30], a 20% reduction in pain (on a numerical rating scale) is clinically relevant. In order to detect a 20% difference compared to usual care (1.2 points, assuming a 2.7-point (45%) reduction in pain score in the TENS self-management group and a 1.5-point (25%) reduction in the usual care group, with pooled SD of 2.6), we would need complete data for 198 patients (99 in each study group), for 90% power at a 5% two-tailed significance level. To take account of a maximum 15% loss of data (this level of follow-up has been achieved in previous similar studies in our Centre), we would need 117 participants per treatment group. We will therefore aim to recruit 120 patients per group; 240 patients altogether.

An independent trial steering group will monitor adherence to interventions and recruitment rates and general progress against the plan.

### Analysis

Descriptive statistics of baseline characteristics will be compared between study groups. An unadjusted analysis will focus on analysing the primary outcome measure (average pain intensity) at 6 weeks (the primary endpoint), using the *t *test for independent samples. Mean estimates and 95% confidence intervals will be presented for differences between groups. The robustness of the findings will be tested through sensitivity analyses (see below). In addition, multilevel analysis will be carried out to estimate the overall effect of TENS treatment over time, taking account of clustering due to repeated measurements on the same individuals. Treatment main effects and the interaction of treatment and time will be modelled for short to long-term outcome data (6 weeks, 6 months and 12 months) and separately for the 2-week post-treatment time period covered by the pain diaries. Differences in secondary outcome measures will be estimated using similar statistical methods. In all cases, a significance level of 5% (two-tailed) is pre-stipulated. Hypothesis testing will be performed blind to treatment group.

#### Sensitivity analysis

The primary analysis will be carried out using an intention to treat (ITT) approach; a sensitivity analysis will use a per protocol approach [31], excluding patients who did not adhere to intervention protocols, received additional treatments during the first 6 weeks (e.g. injection or physiotherapy), or dropped out of the study. Analysis will be carried out in two ways: (a) through a complete-case analysis, i.e. only participants with complete outcome data will be analysed; (b) through an imputed-case analysis, i.e. the multiple imputation method will be used to impute values for missing data (assumed to be missing at random) [32].

A second sensitivity analysis will adjust for the following baseline covariates: age, gender, pain intensity (primary outcome measure) and corresponding baseline value for secondary outcome measures, if applicable; e.g. baseline function scores for the analysis involving limitation in function during follow-up.

#### Process evaluation

Descriptive statistics will be used to describe beliefs and expectations regarding treatment, compliance to advice regarding use of analgesics and use of the TENS units, and satisfaction with treatment. Compliance with TENS will be ascertained from questions about the duration, frequency, and dosage of use as recorded by the patient (in the two-week diary and follow-up questionnaire at 6 weeks). In exploratory subgroup analyses we will analyse whether better outcomes in key outcome measures are associated with:

• older/younger patients

• female/male sex

• manual/non-manual workers

• higher baseline expectations of or beliefs in TENS

• higher scores on satisfaction

• better compliance with advice or use of TENS.

#### Economic evaluation

The economic analysis will adopt a societal perspective, where all relevant costs are measured, including direct health care costs incurred within both public and private sectors, and the indirect costs outside the health care sector associated with productivity loss. A cost-effectiveness analysis will be carried out with change in pain intensity as the clinical outcome of interest. Additionally, a cost-utility analysis will be performed to enable comparisons to be drawn with other areas of health care; utility will be measured using QALY values, which will be derived separately from (i) the EuroQoL EQ5D, and (ii) the SF-12 SF-6D. The ceiling QALY will be 1, which is equivalent to the year's follow up spent in full health.

Data on health care resource use and time off work will be obtained from the patient questionnaires; a medical record review will be used to verify the self-complete data. NHS care will be costed as national averages [33], with inpatient and outpatient episodes costed using NHS reference costs. Owing to the paucity of high-quality unit cost data for private health care consultations, these data will be costed as the NHS equivalent. The British National Formulary will be used to cost prescribed medication. Costs of absenteeism from paid work will be estimated by multiplying the reported number of days off work during the 12-month follow up period by the average daily wage, and stratifying by hourly mean income according to sex, full/part-time work status, and standard occupational classification (SOC 2000) [34,35].

Cost data alongside trials are invariably skewed. We will calculate 95% confidence intervals around differences in mean costs, EQ-5D scores, QALYs, and pain change scores using conventional parametric methods and bias-corrected and accelerated (BCa) bootstrapping (1000 replications) [36]. The aim of the economic evaluation will be to estimate and compare the additional costs of TENS compared to usual primary care only and relate this to the additional effects of TENS. An incremental approach will be used in the analysis if neither treatment group is dominant, with differences in costs and health outcomes expressed using an incremental cost per 1-point improvement in pain intensity (i.e. the incremental cost-effectiveness ratio [ICER]). Similarly, for the cost-utility analysis we will analyse the incremental cost per QALY (i.e. via the incremental cost-utility ratio [ICUR]). Estimates of ICER and ICUR will be derived and bootstrap samples will be generated to compose cost utility planes (which show graphically the variability in the data) [37]. Cost effectiveness acceptability curves (CEACs) will be plotted to quantify, from the bootstrap data, the probabilities of the interventions being cost effective across a range of ceiling ICER values (otherwise referred to as the willingness-to-pay threshold values) [38].

An independent data monitoring committee will monitor the trial every 6 months. There will be no interim analyses.

## Ethical Approval

Ethical approval was obtained from the South Staffordshire Local Research Ethics Committee in May 2009 (ref number 09/H1203/31).

## Discussion

The TATE trial will investigate the clinical and cost effectiveness of TENS in addition to usual primary care management for patients with tennis elbow. TENS may prove to be a suitable intervention for tennis elbow as it may help to reduce pain in the early stages of the condition, without the risk of side effects and/or recurrence in the long-term, and is an attractive treatment option as it facilitates self-management of the condition.

We estimate that we will recruit 240 patients to the trial in 15 months if we involve 20-30 GP practices from north Staffordshire PCT. Trial recruitment is scheduled to begin in August 2009. Follow up is targeted for completion by November 2011 and results should be finalized for publication in spring 2012.

## Competing interests

The authors declare that they have no competing interests.

## Authors' contributions

All authors participated in the design of the trial and the drafting of the manuscript. All authors have read and approved the final manuscript.

## Pre-publication history

The pre-publication history for this paper can be accessed here:

http://www.biomedcentral.com/1471-2474/10/156/prepub

## References

[B1] Smidt N Lewis M Windt DA van der Hay EM Bouter LM Croft P Lateral epicondylitis in general practice: course and prognostic indicators of outcome J Rheumatol 2006 33 2053 2059 16881095

[B2] Hamilton PG The prevalence of humeral epicondylitis: a survey in general practice J R Coll Gen Pract 1986 36 464 465 3440991 PMC1960610

[B3] Dimberg L The prevalence and causation of tennis elbow (lateral humeral epicondylitis) in a population of workers in an engineering industry Ergonomics 1987 30 573 579 10.1080/00140138708969746 3595554

[B4] Smidt N Assendelft WJ Arola H Malmivaara A Greens S Buchbinder R Windt DA van der Bouter LM Effectiveness of physiotherapy for lateral epicondylitis: a systematic review Ann Med 2003 35 51 62 10.1080/07853890310004138 12693613

[B5] Trudel D Duley J Zastrow I Kerr EW Davidson R Macdermid JC Rehabilitation for patients with lateral epicondylitis: a systematic review J Hand Ther 2004 17 243 266 10.1197/j.jht.2004.02.011 15162109

[B6] Bisset L Paungmali A Vicenzino B Beller E A systematic review and meta-analysis of clinical trials on physical interventions for lateral epicondylalgia Br J Sports Med 2005 39 411 422 10.1136/bjsm.2004.016170 15976161 PMC1725258

[B7] Bisset L Beller E Jull G Brooks P Darnell R Vicenzino B Mobilisation with movement and exercise, corticosteroid injection, or wait and see for tennis elbow: randomised trial BMJ 2006 333 939 10.1136/bmj.38961.584653.AE 17012266 PMC1633771

[B8] Hay EM Paterson SM Lewis M Hosie G Croft P Pragmatic randomised controlled trial of local corticosteroid injection and naproxen for treatment of lateral epicondylitis of elbow in primary care BMJ 1999 319 964 968 10514160 10.1136/bmj.319.7215.964 PMC28251

[B9] Smidt N Windt DA van der Assendelft WJ Devillé WL Korthals-de Bos IB Bouter LM Corticosteroid injections, physiotherapy, or a wait-and-see policy for lateral epicondylitis: a randomised controlled trial Lancet 2002 359 657 662 10.1016/S0140-6736(02)07811-X 11879861

[B10] Lewis M Hay EM Paterson SM Croft P Local steroid injections for tennis elbow: does the pain get worse before it gets better? Results from a randomized controlled trial Clin J Pain 2005 21 330 334 10.1097/01.ajp.0000125268.40304.b3 15951651

[B11] Johnson MI Transcutaneous Electrical Nerve Stimulation (TENS) and TENS-like devices: do they provide pain relief? Pain Rev 2001 8 121 158 10.1191/0968130201pr182ra

[B12] Johnson M Martinson M Efficacy of electrical nerve stimulation for chronic musculoskeletal pain: a meta-analysis of randomized controlled trials Pain 2007 130 157 165 10.1016/j.pain.2007.02.007 17383095

[B13] Bjordal JM Johnson MI Lopes-Martins RA Bogen B Chow R Ljunggren AE Short-term efficacy of physical interventions in osteoarthritic knee pain. A systematic review and meta-analysis of randomised placebo-controlled trials BMC Musculoskelet Disord 2007 8 51 10.1186/1471-2474-8-51 17587446 PMC1931596

[B14] Brosseau L Judd MG Marchand S Robinson VA Tugwell P Wells G Yonge K Transcutaneous electrical nerve stimulation (TENS) for the treatment of rheumatoid arthritis in the hand Cochrane Database Syst Rev 2003 3 CD004377 12918009 10.1002/14651858.CD004377 PMC8826159

[B15] Bjordal JM Johnson MI Ljunggreen AE Transcutaneous electrical nerve stimulation (TENS) can reduce postoperative analgesic consumption. A meta-analysis with assessment of optimal treatment parameters for postoperative pain Eur J Pain 2003 7 181 188 10.1016/S1090-3801(02)00098-8 12600800

[B16] Johnson MI Aston CH Thompson JW Long term use of transcutaneous electrical nerve stimulation at Newcastle Pain Relief Clinic J R Soc Med 1992 85 267 268 1433087 10.1177/014107689208500508 PMC1294602

[B17] Fishbain DA Chabal C Abbott A Heine LW Cutler R Transcutaneous electrical nerve stimulation (TENS) treatment outcome in long-term users Clin J Pain 1996 12 201 214 10.1097/00002508-199609000-00008 8866161

[B18] Chesterton LS Barlas P Foster NE Lundeberg T Wright CC Baxter GD Sensory stimulation (TENS): effects of parameter manipulation on mechanical pain thresholds in healthy human subjects Pain 2002 99 253 262 10.1016/S0304-3959(02)00118-5 12237203

[B19] Chesterton L Foster NE Wright CC Baxter GD Barlas P Effects of TENS frequency, intensity and stimulation site parameter manipulation on pressure pain thresholds in healthy human subjects Pain 2003 106 73 80 10.1016/S0304-3959(03)00292-6 14581113

[B20] Khadilkar A Milne S Brosseau L Robinson V Saginur M Shea B Tugwell P Wells G Transcutaneous electrical nerve stimulation (TENS) for chronic low-back pain Cochrane Database Syst Rev 2005 3 CD003008 16034883 10.1002/14651858.CD003008.pub2

[B21] Halle JS Franklin RJ Karalfa BL Comparison of four treatment approaches for lateral epicondylitis J Orthop Sports Phys Ther 1986 8 62 69 18802239 10.2519/jospt.1986.8.2.62

[B22] Weng CS Shu SH Chen CC Tsai YS Hu WC Chang YH The evaluation of two modulated frequency modes of acupuncture-like TENS on the treatment of tennis elbow pain Biomed Eng - Appl Basis Commun 2005 17 236 242 10.4015/S1016237205000354

[B23] Hay EM Foster NE Thomas E Peat G Phelan M Yates HE Blenkinsopp A Sim J Effectiveness of community physiotherapy and enhanced pharmacy review for knee pain in people aged over 55 presenting to primary care: pragmatic randomised trial BMJ 2006 333 995 10.1136/bmj.38977.590752.0B 17056608 PMC1635605

[B24] Bot SDM Terwee CB Windt DA van der Feleus A Bierma-Zeinstra SM Knol DL Bouter LM Dekker J Internal consistency and validity of a new physical workload questionnaire Occup Environ Med 2004 61 980 986 10.1136/oem.2003.011213 15550603 PMC1740683

[B25] Karasek RA Pieper CF Schwartz JE Job Content Questionnaire and User's Guide: revision 1 1985 Los Angeles: USCLA

[B26] Moss-Morris R Weinman J Petrie K Horne R Cameron L Buick D The Revised Illness Perception Questionnaire (IPQ-R) Psychol Health 2002 17 1 16 10.1080/08870440290001494

[B27] Overend TJ Wuori-Fearn JL Kramer JF MacDermid JC Reliability of a patient-rated forearm evaluation questionnaire for patients with lateral epicondylitis J Hand Ther 1999 12 31 37 10192633 10.1016/s0894-1130(99)80031-3

[B28] EuroQolGroup EuroQol - a new facility for the measurement of health-related quality of life Health Policy 1990 16 199 208 10.1016/0168-8510(90)90421-9 10109801

[B29] Brazier JE Roberts J The estimation of a preference-based measure of health from the SF-12 Med Care 2004 42 851 859 10.1097/01.mlr.0000135827.18610.0d 15319610

[B30] Farrar JT Young JP Jr LaMoreaux L Werth JL Poole RM Clinical importance of changes in chronic pain intensity measured on an 11-point numerical pain rating scale Pain 2001 94 149 158 10.1016/S0304-3959(01)00349-9 11690728

[B31] Wright CC Sim J Intention to treat approach to data from randomized controlled trials: a sensitivity analysis J Clin Epidemiol 2003 56 833 842 10.1016/S0895-4356(03)00155-0 14505767

[B32] Schafer JL Multiple imputation: a primer Stat Methods Med Res 1999 8 3 15 10.1191/096228099671525676 10347857

[B33] Curtis L Netten A Unit Costs of Health and Social Care: 2004 2004 Canterbury: Personal Social Services Research Unit, University of Kent

[B34] Office of National Statistics Standard Occupational Classification 2000: The coding index 2000 London: The Stationery Office

[B35] Office for National Statistics Annual survey of hours and earnings (ASHE). Analysis by Occupation 2004 London: The Stationery Office

[B36] Mullner M Commentary: Bootstrapping simplifies appreciation of statistical inferences BMJ 2003 326 914 12714463 10.1136/bmj.326.7395.900/b

[B37] Korthals-de Bos I van Tulder M van Dieten H Bouter L Economic evaluations and randomized trials in spinal disorders: principles and methods Spine 2004 29 442 448 10.1097/01.BRS.0000102683.61791.80 15094541

[B38] Fenwick E Claxton K Sculpher M Representing uncertainty: the role of cost-effectiveness acceptability curves Health Econ 2001 10 779 787 10.1002/hec.635 11747057

